# A New Approach to Detecting and Measuring Changes in the Feeding Behaviour Habits of Group-Housed Growing–Finishing Pigs

**DOI:** 10.3390/ani12121500

**Published:** 2022-06-09

**Authors:** Marta Fornós, Mercè Farré, Sergi López-Vergé, Encarnación Jiménez-Moreno, Vicente Rodríguez-Estévez, Josep Gasa

**Affiliations:** 1 Cargill Animal Nutrition, Mequinenza, 50170 Zaragoza, Spain; encarnacion_jimenez@cargill.com; 2 Animal Nutrition and Welfare Service, Department of Animal and Food Sciences, Universitat Autònoma de Barcelona, 08193 Bellaterra, Spain; sergilv1@gmail.com (S.L.-V.); josep.gasa@uab.cat (J.G.); 3 Department of Mathematics, Area of Statistics and Operations Research, Universitat Autònoma de Barcelona, 08193 Bellaterra, Spain; merce.farre@uab.cat; 4 Department of Animal Production, Universidad de Córdoba, 14071 Córdoba, Spain; pa2roesv@uco.es

**Keywords:** feeding behaviour habits repeatability, growing-finishing pigs, environmental conditions, physical feed form

## Abstract

**Simple Summary:**

It is known that feeding behaviour habits (FBHs) influence growing–finishing pigs’ performance and are modified by production conditions on a group scale. However, no methods are available to describe the evolution of the FBHs at the individual level over short- or long-term periods. This work presents two methods, i.e., repeatability (ratio between animal and total variance) and a new non-parametric approach named “maintenance”, which provides information at the individual level by typifying the individuals into classes and quantifying (%) the pigs that show similar FBHs. Both concepts were applied to six consecutive 14-day periods, throughout two trials of group-housed growing–finishing pigs under different environmental conditions and fed different physical feed forms (mash and pelleted). Both repeatability and maintenance indexes showed similar but not identical results, since they provide complementary information at the global and individual levels, respectively. The results indicate that the environmental conditions and physical feed form modified the repeatability and maintenance of most FBHs, except for the average daily feed intake. Moreover, since maintenance provides data at the individual level, this may be an interesting approach in further studies, for example, to analyse the influence of pen hierarchy on FBHs.

**Abstract:**

The present work aims to estimate the methods of repeatability and of a new non-parametric approach based on typifying individuals into classes and quantifying (%) the pigs in a group that show similar feeding behaviour habits (FBHs) in consecutive periods (“maintenance”). Both methods were estimated over six consecutive 14-day periods in two trials of group-housed growing–finishing pigs (*n* = 60 each). The first trial started in summer and ended in autumn, and pigs were fed a pelleted diet (HT-P). The second trial started in spring and ended in summer, and the same diet was fed mash (TH-M). The average daily feed intake obtained the lowest repeatability and maintenance values, and it progressively decreased as pigs grew, independent of environmental conditions or physical feed form, whereas the maintenance and repeatability of the number of feeder visits and the visit size decreased when environmental conditions changed from temperate to hot, and mash-fed pigs had higher maintenance and repeatability values for the time spent eating than pellet-fed pigs. In conclusion, the new approach (maintenance) is a tool that is complementary to the classic repeatability concept and is useful for analysing the evolution of FBHs across periods of time at the individual level.

## 1. Introduction

Feeding behaviour habits (FBHs) influence the performance and carcass quality of growing–finishing pigs [1,2,3,4]. External factors such as environmental conditions [5,6], physical feed form [7] or feeder competition [8] modify FBHs. Under hot conditions, growing–finishing pigs eat their daily feed intake (DFI) in two accentuated daily peaks [9], reduce daily feeder visits (TVs), time spent eating (TD) and meal size (MS) with no differences in feeding rate (FR) compared with pigs reared under thermoneutral conditions [5,6]. On the other hand, pigs fed mash spend more time eating due to lower FR compared with pigs fed pellets [7,10]. Similarly, when pigs’ diet depends on foraging natural resources, pigs eat slowly and spend more time grazing with two daily intervals separated by a resting period [11]. Moreover, when the access to the feeder is disrupted, social pressure increases, and pigs change their FBHs in order to achieve their DFI [12]. However, no data exist regarding whether those factors influence all the pigs within a pen equally. Therefore, knowledge about the evolution of FBHs as pigs grow, together with the effect of some production conditions on FBHs at the individual level, is of interest.

The repeatability coefficient [13] informs us about the FBH permanence of a group of animals over specific periods. In fact, De Haer and Merks [14] and Hyun et al. [15] computed the repeatability estimates in group-housed growing–finishing pigs and found the lowest repeatability values (<0.3) for the average daily feed intake (ADFI), both over short (2 weeks) and longer periods (from 25 to 100 kg BW and from 27 to 80 kg BW pigs, respectively). Over shorter periods, both authors found medium–high repeatability values for TVs, TD, MS and FR (between 0.35 and 0.61). However, over longer periods, they found lower and more variable values. Moreover, since repeatability measures FBHs at the group level, and large variability among individuals has been reported [4], it is of interest to develop a complementary approach to characterize which individuals change their FBHs as they grow. This approach may help to analyse if all the pigs of a pen modify their FBHs under specific external changes or if the hierarchy within a pen influences FBHs. In fact, high-ranking animals perform fewer and larger feeder visits compared with low-ranking pigs [16]. Therefore, the individual identification of the pigs that maintain or do not maintain their FBHs over time and under different external conditions proves to be of interest. 

Consequently, the objectives of the present work are (1) to describe how to estimate FBHs repeatability, (2) to define and compute a new complementary concept, named “maintenance”, and (3) to calculate and analyse both indicators, repeatability and maintenance, throughout the growing–finishing period of grouped-housed pigs in two trials conducted under different environmental conditions and using different physical feed forms (mash and pellets).

## 2. Materials and Methods

### 2.1. Experimental Approach Design 

Two growing–finishing pig trials were conducted to analyse the repeatability and the maintenance of FBHs. Both trials were conducted on the same farm located in the northeast of Spain in two consecutive years (2018 and 2019). In each trial, the first 30 days of fattening were used as a period of adaptation to the automatic feeding system. After this first period, FBHs were analysed for 84 days divided in six periods of 14 days each (p1–p6) from day 31 to day 114 of fattening. Both trials were carried out under commercial conditions, and the environmental conditions of the trials differed, although the temperature was regulated by open-air ventilation with automatic temperature-probe-controlled curtains. The first trial started in June with an average temperature of 30.7 °C on day 31 of fattening (the first three periods were classified as hot season) and finished in October with an average temperature of 21.2 °C on day 114 (the last three periods were classified as temperate season), and pigs were fed pelleted feed (Hot Temperate/Pelleted trial; HT-P). The second trial started in March with an average temperature of 22.8 °C on day 31 of fattening (the first three periods were classified as temperate season) and finished in July with an average temperature of 29.4 °C on day 114 (the last three periods were classified as hot season), and pigs were fed the same diet in mash form (Temperate Hot/Mash trial; TH-M). The experimental unit for all the analyses was the pig. 

### 2.2. Animals and Housing Conditions

A total of 60 crossbred Pietrain × (Landrace × Large White) pigs of 60 ± 3 days old and coming from the same nursery facility were used in each trial. On arrival, pigs were split into three initial body weight (BW) groups, and four pigs in each BW group were allotted to each pen of 12 pigs at random (16.5 ± 0.91 and 17.9 ± 0.72 kg (mean ± SD) for HT-P and TH-M pigs, respectively). Five pens per trial were used (Table 1).

Each pen (12 m^2^) was equipped with an automatic feeding system (Nedap ProSense^®^; Groenlo, The Netherlands), one nipple with a water cup, totally slatted concrete floor and open-air ventilation with automatic temperature-probe-controlled curtains. An enrichment item (pieces of wood at the end of chains) categorised as a suboptimal material was provided in each pen [17]. The stocking density per pen was 0.91 m^2^/pig (excluding the space occupied by the automatic feeding system), which is above the minimum space per pig set by European legislation 2008/120/EC [18]. Pigs were given ad libitum access to water and feed during all the experimental periods. Five pigs were discarded during the experimental periods: two pigs due to lameness problems in the HT-P trial and three pigs, one due to recording-system problems and two due to respiratory disorders, in the TH-M trial. Hence, the final data regarded 58 pigs for the HT-P trial and 57 pigs for the TH-M trial. 

To evaluate the environmental conditions, the indoor relative humidity and temperature were measured every ten minutes throughout the experimental period with a data logger testo 175 H1 (Testo SE & Co. KGaA^®^, Titisee-Neustadt, Germany). The daily average temperature-humidity index (THI) of the experimental period was calculated using the equation proposed by Lallo et al. [19], where T = temperature in °C, and RH = relative humidity in percentage:

THI (°C)=Tmax(°C)−(0.55−(0.0055RH)(Tmax(°C)−14.5))

From the THI results, four categories of heat stress were defined according to Marai et al. [20]: normal (THI < 27.8), moderate (THI between 27.8 and 28.8), severe (THI between 28.9 and 29.9) and emergency zone (THI ≥ 30).

In both trials, the same common diet in a 3-phase feeding program was fed; feeding programs differed only in the physical feed form, being pellets in the HT-P trial and mash in the TH-M trial. The diets, based on wheat-corn and soybean meal, were formulated to fulfil the FEDNA [21] requirements.

### 2.3. Feeding Behaviour Habits

On the first day of the pre-experimental period, after allotting the pigs into the pens, all pigs were individually identified with an electronic full duplex ear tag, which permitted control access to the automatic feeding system by providing a unique identifier for each pig through the radio frequency identification marker located on the tag. With this individual identification, the pig’s code, date, time, feed intake and BW of each feeder visit were recorded. The BW of the pigs was recorded by a scale that weighed the front and back parts of the pig placed in front of the feeder during each feeder visit. The mean BW of the pigs at the start of p1 (day 31 of fattening) was 42.3 ± 4.06 and 41.4 ± 4.81 kg for the HT-P and TH-M trials, respectively (*p* = 0.24). The registered data allowed us to calculate: (1) individual daily feed intake (DFI; kg/d), (2) total feeder visits (TVs; total number of feeder visits per pig per day; visits/d), (3) total visit duration (TD; time spent eating per pig and day; min/d), (4) feeder visit size (VS; amount of feed intake per feeder visit, calculated as DFI/TVs; kg/visit) and (5) feeding rate (FR; g of feed intake per minute spent eating, calculated as (DFI*1000)/TD; g/min). Calculations were performed for the six experimental periods of 14 days each from days 31 to 114 of the growing–fattening period for both trials (HT-P and TH-M). The average of each FBH per pig per period was calculated. In each trial, the changes in the FBHs of two consecutive periods (from p1 to p6) were calculated using two kinds of indicators, repeatability and maintenance, as explained below. 

#### 2.3.1. Feeding Behaviour Habits’ Repeatability

Linear mixed effects models, also named components of variance models and random intercept models in the literature, provide a useful framework for understanding different sources of biological variation and for quantifying the reproducibility of measurements in different periods. After fitting the underlying model, repeatability (RA) is defined as the ratio of between-subject component variance to the total residual variance [13,15]. In the present study, RA was computed for each FBH *y* (ADFI, TVs, TD, VS and FR on a logarithmic scale) in six pairs of consecutive periods. Natural logarithms were applied to reduce asymmetry and heavy low tails, as the log-scaled data adjust better to the Gaussian distribution, as noted by Hyun et al. [15].

The model equation is *y_it_ = β_0_ + β_1_p_t_ + ụ_i_ + ε_it_*, where *y*_it_ is the log-scaled FBH measured for individual *i* in period *t*; *p_t_* is a binary indicator (0 if *t = j* and 1 if *t = j + 1);* *u_i_* are assumed to be random-centred Gaussian variables with variance σ^2^_u_ representing a between-subject component; and *ε_it_* are random-centred Gaussian variables with variance σ^2^ representing a within-subject component. It is assumed that all random variables are independent of each other. Box plots and qq plots were applied to the data before and after the logarithmic transformation to check the improvement, and the normality and homogeneity of residual variances were checked using the Shapiro–Wilks and Levene’s tests, respectively.

In this frame, the repeatability was estimated as:

$$\mathrm{RA}=\frac{\sigma_{u}^{2}}{\sigma_{u}^{2}+\sigma^{2}}$$

Thus, RA is the proportion of the total variance due to the variability between individuals and is an intra-class correlation [22] taking values between 0 and 1. The rpt function of the rptR R-library was used in this case [23,24]. Notice that the RA index above is defined by means of a random intercept model which includes time (period) as a two-level factor. It agrees with the named “consistency” repeatability index in the work by Biro and Stamps [25]. Another index, called “conditional” repeatability [25], could be computed on a random intercept and slope model, allowing a different regression line for each individual to be obtained in this way. Because the present longitudinal analysis is reduced to five pairs of consecutive periods, and a specific model is adjusted for each given pair and physical feed form, the random and slope–intercept model should be discarded. In fact, these models produce a perfect fit for any pair of consecutive measurements, so the residual variance estimate is zero, and the repeatability index equals to one anywhere. In any case, all these underlying models assume a constant residual variability over time, which is not always sustainable and is a drawback of this methodology. The next point is an alternative, assumption-free attempt to estimate repeatability in the broadest sense, that is, the extent to which individual differences in FBHs persist over time.

#### 2.3.2. Feeding Behaviour Habits’ Maintenance

The second developed procedure also aims at measuring and comparing the changes in FBHs but offers a different point of view of the same data, since it does not imply adjusting any model or imposing constraints. The method has two steps; the first one consists of giving a characterization of the pigs that show similar FBHs in two different periods; the second step consists of computing the percentage of pigs in this category, which is called maintenance.

In order to clarify what the statement “a pig maintains or not a determinate FBH in two consecutive periods” means, taking into account that descriptive means and standard deviations change from one period to another, it is proposed to use data standardisation (z-scores). Starting from log-scaled data (*y_it_*) corresponding to a given FBH, the z-score is calculated as usual, centring with respect to the trial mean and dividing by the trial standard deviation:

$$z_{it}=\frac{y_{it}-\mu_{t}\;}{\sigma_{t}}$$

It should be noted that, to exclude excessive influential observations, the sample mean and the standard sample deviation are *trimmed* [26]. It follows that for each variable, period and trial, mean and standard deviation are estimated on the subset of values in the range *Md ± 2IQR*, where *Md* is the median, and *IQR* is the interquartile range; then, z-scores are computed for the whole sample values. z-scores are interpreted as the numbers of standard deviations from the mean of the observation. Each *z*-score explains the position of the pig in the group in response to *y*, specifying the number of standard deviations by which the response of this animal deviates from the mean to the right (+) or to the left (−). As z-scores are unit-free, they may be compared even if they come from different periods.

Consequently, a pig maintains its FBHs during two consecutive periods at the level of a pre-set value δ, if the distance between the two consecutives z-scores is less than δ:

$$│z_{it}\;–\;z_{it+1}│\leq \;\delta .$$

Then, maintenance is defined as the percentage of pigs in the trial that satisfy this condition. 

The above inequality expresses a condition on the distance between two standard punctuations, a particular case of Mahalanobis distance [27] that is defined on multivariate measures, and the particular univariate case was considered here. Maintenance results depend monotonically on threshold δ. The smaller δ is, the more restrictive is the maintenance condition; therefore, the smaller the maintenance values are. In the present work, after calculating different δ values, δ = 0.75 was used as it fitted the profiles of the repeatability estimates of the FBHs of growing–finishing pigs as they grew. Consult the appendix A for our statistical arguments in relation to this point. An example of usage and some theoretical considerations around threshold δ = 0.75 are discussed in the Appendix A. 

## 3. Results

### 3.1. Environmental Conditions

In the HT-P trial, the median THI values of p1, p2 and p3 were above the THI emergency level (THI > 30), whereas in p4, p5 and p6, the median THI values were below the moderate THI level (<28.8). In the TH-M trial, the median THI values in p1, p2 and p3 were below the normal THI level (<27.8), whereas in p4, the median THI value was in the moderate level (between 27.8 and 28.8), and in p5 and p6, the median THI values were above the THI emergency level (THI > 30). The THI levels obtained suggest that pigs were under heat stress during p1-p2-p3 and p4-p5-p6 of the HT-P and TH-M trials, respectively, and these were the periods named hot seasons; on the other hand, during p4-p5-p6 and p1-p2-p3 of the HT-P and TH-M trials, respectively, pigs were under thermoneutral conditions, and both sets of periods were named temperate seasons. Moreover, the average temperature for each season was calculated, being 28.0 and 25.5 °C for the hot and temperate seasons, respectively, of the HT-P trial and 23.5 and 27.3 °C for the temperate and hot season, respectively, of TH-M. The highest sudden changes in THI and temperature in both trials happened between p3 and p4.

### 3.2. Main Descriptive Statistics of Feeding Behaviour Habits Per Period and Trial

Table 2 includes a summary of the main descriptive statistics of FBHs per period and trial (expressed as mean). HT-P pigs performed a greater number of TVs than TH-M pigs throughout the periods studied, together with a larger decrease from p1 to p6 compared with the TH-M trial (52% vs. 24%, respectively). HT-P pigs spent less time eating than TH-M pigs in all the periods analysed, and the reduction in time was greater in the HT-P trial (32% vs. 26%, respectively). VS increased as pigs grew in both trials with increases of 214% and 103% in the HT-P and TH-M trials, respectively. HT-P pigs had a higher FR than TH-M pigs in all the periods analysed with increases of 121% and 114% in the HT-P and TH-M trials, respectively. However, to calculate the maintenance, the trimmed mean and trimmed standard deviation at a log-scale of each FBH analysed (TVs, TD, VS and FR) by period are needed, and these are shown in Table 3.

### 3.3. Repeatability and Maintenance of ADFI

**Figure 1:**
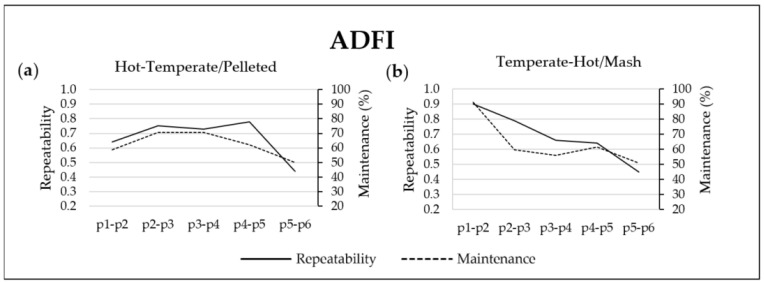
Repeatability (left axis) and maintenance (right axis) of the average daily feed intake (ADFI) during two consecutive 14-day periods from p1 to p6 (six periods of 14 days each from day 31 to day 114 of fattening) and by trial: (**a**) Hot-Temperate/Pelleted trial and (**b**) Temperate-Hot/Mash trial. The average temperature on day 31 and on day 114 of fattening was of 30.7 and 21.2 °C in the Hot-Temperate/Pelleted trial and of 22.8 and 29.4 °C in the Temperate-Hot/Mash trial, respectively.

Figure 1 shows the repeatability and maintenance values of the ADFI in both trials and between consecutive periods. In the HT-P trial (Figure 1a), ADFI repeatability and maintenance showed a similar evolution during the experimental period. From p1–p2 to p2–p3, the repeatability and maintenance values of the ADFI increased from 0.64 to 0.75 and from 59 to 71%, respectively. From p3–p4 to p5–p6, the repeatability and maintenance values of the ADFI decreased progressively from 0.73 to 0.44 and from 71 to 50%, respectively. In the TH-M trial (Figure 1b), ADFI repeatability and maintenance values decreased progressively from 0.90 to 0.45 and from 91 to 51%, respectively.

### 3.4. Repeatability and Maintenance of TVs, TD, VS and FR in the Hot-Temperate/Pelleted Trial

**Figure 2:**
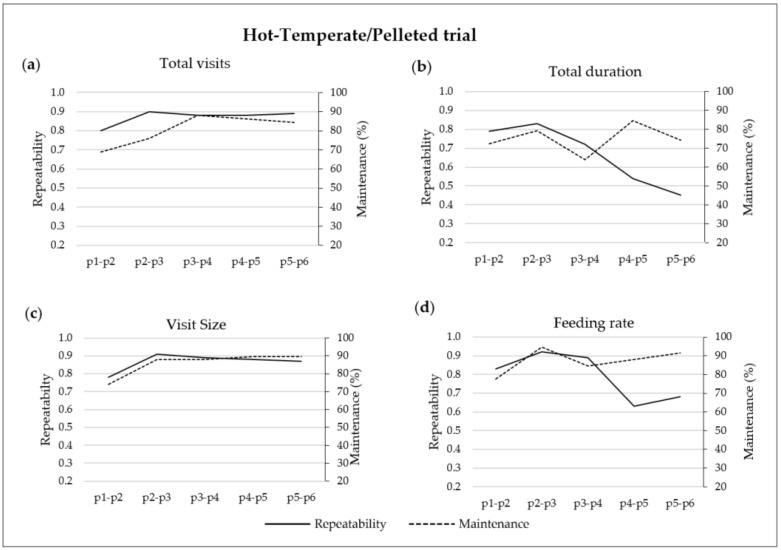
Repeatability (left axis) and maintenance (right axis) of (**a**) total visits, (**b**) total duration, (**c**) visit size and (**d**) feeding rate over two consecutive 14-day periods from p1 to p6 (six periods of 14 days each from day 31 to day 114 of fattening) in the Hot-Temperate/Pelleted trial (the average temperatures were of 30.7 and 21.2 °C on day 31 and on day 114 of fattening).

The repeatability and maintenance values between consecutive periods of TVs, TD, VS and FR of the HT-P trial are shown in Figure 2. TVs repeatability and maintenance increased gradually throughout the experimental period from 0.80 to 0.89 and from 69 to 85%, respectively (Figure 2a). The repeatability of TD started at 0.79 from p1 to p2, increased to 0.83 from p2 to p3 and progressively decreased to 0.45 from p5 to p6. On the other hand, in terms of maintenance, the lowest value was obtained from p3 to p4, with 64% of the pigs maintaining their TD, and the highest value was obtained from p4 to p5, with 85% of the pigs maintaining their TD between both periods (Figure 2b). VS repeatability and maintenance values showed a parallel progressive increase throughout the experimental period from 0.78 to 0.87 and from 74% to 89%, respectively (Figure 2c). The evolution of the repeatability and maintenance of FR also made similar progress until p4, starting with the values of 0.83 and 78% from p1 to p2 and increasing up to 0.92 and 95% from p2 to p3, respectively. In contrast, from p4 to p5 and from p5 to p6, the repeatability values decreased to 0.63 and 0.68; meanwhile, maintenance increased to 88 and 91%, respectively (Figure 2d).

### 3.5. Repeatability and Maintenance of TVs, TD, VS and FR in the Temperate-Hot/Mash trial

**Figure 3:**
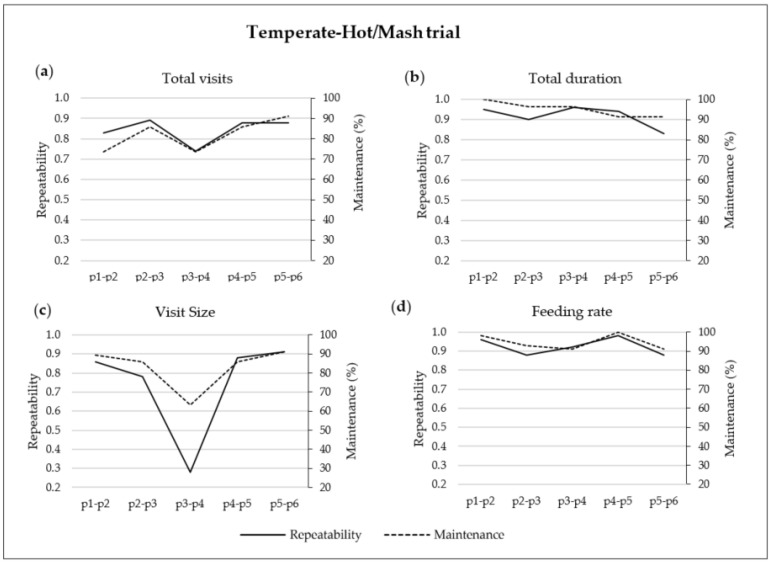
Repeatability (left axis) and maintenance (right axis) of (**a**) total visits, (**b**) total duration, (**c**) visit size and (**d**) feeding rate over two consecutive 14-day periods from p1 to p6 (six periods of 14 days each from day 31 to day 114 of fattening) in the Temperate-Hot/Mash trial (the average temperatures were of 22.8 and 29.4 °C on day 31 and on day 114 of fattening).

The repeatability and maintenance values of TVs, TD, VS and FR over consecutive periods in the Temperate-Hot/Mash trial are shown in Figure 3. The repeatability and maintenance of TVs started from p1 to p2 with a repeatability of 0.83 and 74% of the pigs maintaining their TVs and increased up to 0.89 and 86% from p2 to p3, respectively. From p3 to p4, TVs repeatability and maintenance decreased to 0.74 and 74% and progressively increased to 0.88 and 91% from p5 to p6, respectively (Figure 3a). The repeatability and maintenance of TD progressively decreased throughout the experimental period from 0.95 to 0.83 and from 100 to 91%, respectively (Figure 3b). The repeatability and the maintenance values of VS were above 0.78 and 85% across all periods, except from p3 to p4, in which they decreased to 0.28 and 63%, respectively (Figure 3c). The repeatability and maintenance values of FR were above 0.88 and 91%, respectively, across all the periods analysed (Figure 3d).

## 4. Discussion

### 4.1. Repeatability vs. Maintenance

The two approaches used to characterize and assess the changes in growing–finishing pigs’ FBHs as they grow, repeatability and maintenance, provide complementary information that is not identical but similar and highly congruent. In fact, the maintenance concept may be defined as an alternative but complementary method to the repeatability ratio. Repeatability describes the FBHs degree of permanence on a group scale. A low repeatability value of a specific FBH in a determined group of animals means that this value is poorly predictable over time. However, this low repeatability result could be due to the fact that most animals in the group have low repeatability values or that a low number of pigs have extremely low repeatability coefficients. The latter was observed from p3 to p4 in the TH-M trial, in which the low repeatability of 0.28 of VS was a consequence of only 37% of the pigs that did not maintain their VS feeding habits across both periods. Therefore, the main contribution to the maintenance concept is that it brings information at the individual level, which makes it an interesting and applicable tool for fields in which it is especially valuable to pay attention to the individuality of animals, such as in the assessment of the welfare of growing–finishing pigs. In fact, Bus et al. [28] observed that reduced welfare is associated with deviations in FBHs. However, those authors reported that a large variability in FBHs among individuals exists and should be well understood before the variation that represents pig welfare can be interpreted, a case in which the use of the maintenance approach is of interest. In addition, feeder space influences the growth performance [29] and FBHs [30] of growing–finishing pigs. However, to our knowledge, no data have been published regarding the influence of feeder space at the individual level, and maintenance may be a useful tool to better understand the relation between feeder space and growth performance. Moreover, the maintenance approach could also be used to better understand the influence of the hierarchy within a pen on the welfare, FBHs and performance of pigs reared under different housing conditions. In the present study, the maintenance approach allowed us to identify which pigs maintained their FBHs and which did not across specific periods, and from this individual characterization, the computation of maintenance as a percentage of pigs that did not change their FBHs provided useful information at the group level. The main advantage of repeatability is that it is a traditional concept [31,32], which is thoroughly used to analyse FBHs in growing–finishing pigs [14,15]. Furthermore, repeatability is calculated by fitting a variance component model [22], which is available in any statistical software. The main drawback is that the method involves fitting a parametric model with constraints (Gaussianity and equal variances, which are difficult to sustain in different periods), to then calculate what proportion of the residual variance is explained by the variability between subjects, which is a fairly abstract notion. Moreover, inference on repeatability is usually based on bootstrapping, which is asymptotically consistent but does not provide general guarantees in small samples. The maintenance procedure is based on intuitive notions and is a non-parametric calculation that does not require any underlying model or any restriction on data distribution. Moreover, percentage values are easy to interpret. However, one drawback is that maintenance calculation depends on a somewhat arbitrary threshold related to the expected evolution of the values of the studied FBHs. In the present study, the selected threshold value was 0.75 (there is a brief technical discussion in Appendix A). 

To sum up, maintenance is a parameter complementary to the more classical repeatability concept; it represents a step forward to identify which percentage of individually growing–finishing pigs maintain their FBHs over time. In addition, the maintenance approach is a new tool that may be used to better understand the effect of social feeding dynamics of animals within a pen.

### 4.2. Production Factors That Modify the Repeatability and Maintenance of Feeding Behaviour Habits

In both experiments, the time evolution of the repeatability and maintenance of most registered FBHs evolved in a parallel shape (see Figure 1, Figure 2 and Figure 3). The repeatability values of the ADFI (Figure 1) were usually the lowest among the evaluated FBHs. Independently of the environmental conditions and physical feed form, ADFI repeatability decreased as the pigs’ BW increased, obtaining the lowest values at the end of the experiments, from p5 to p6 (0.45). Previous findings also reported the lowest repeatability values for the ADFI [14,15]. Those studies found values of 0.21, 0.27 and 0.26 over 10-, 5- and 2-week periods, respectively [15], and 0.09, 0.14 and 0.29 from 25 to 100 kg BW over 4- and 2-week periods, respectively [14]. The decrease in the repeatability values obtained in the present study as pigs grew was also observed in the maintenance values, which started with 74% of the pigs maintaining their ADFI feeding habits from p1 to p2 and decreased to 50% from p5 to p6. Those results are in line with the findings obtained by Carcò et al. [4], who observed a higher variability in the ADFI of older pigs than in that of younger pigs. Therefore, it follows that as pigs grow, the individual predictability of the ADFI becomes more difficult.

Environmental conditions and physical feed form influence the FBHs of growing–finishing pigs under commercial conditions [5,6,7]; consequently, they could modify their repeatability and maintenance values. In the present study, it was impossible to separate the effects of environmental conditions and physical feed form on FBHs. However, it is still interesting to discuss the changes in the repeatability and maintenance of the FBH parameters obtained in both experiments separately in order to compare and explain the value of the data generated by both approaches together.

For instance, hot conditions jeopardize the performance results and modify the FBHs of growing–finishing pigs [5,6,33,34]. However, the bibliography does not illustrate the impact of environmental conditions on the repeatability and maintenance of the FBHs of growing–finishing pigs. In the present study, small changes in terms of FBHs were observed when environmental conditions changed from hot to temperate (HT-P trial, from p3 to p4). In fact, the only FBH affected was TD, with 36% of the pigs changing their TD. On the other hand, when the environmental conditions changed from temperate to hot in the TH-M trial (from p3 to p4), the repeatability of TVs and VS decreased to 0.74 and 0.28, due to the fact that 26 and 37% of the pigs modified their TVs and VS feeding habits, respectively. TD and FR repeatability and maintenance were not affected (>0.83 repeatability and > 91% maintenance values). Furthermore, the changes in the TVs and VS feeding habits were maintained during the hot season with repeatability values above 0.88 and maintenance values above 85% (TH-M trial, from p4 to p6), suggesting a form of FBHs adaptation. The observed TVs and VS changes agree with the findings obtained by Quiniou et al. [5], who observed that group-housed 63 kg BW pigs reduced their TVs and MS and maintained their FR with an increase in the temperature from 19 to 29 °C; however, they also found changes in TD. 

Although the effect of physical feed form on repeatability and maintenance is cofounded by the differences in environmental conditions, pigs fed mash obtained higher TD repeatability and maintenance values (>0.83 and >91%, respectively) than pigs fed pellets (<0.83 and <85%, respectively) for all the periods analysed. According to Laitat et al. [10] and Li et al. [7], mash-fed pigs eat more slowly; consequently, they need more time to reach the desired DFI than pellet-fed pigs, which could be the cause of the higher feeder occupancy rate in mash-fed pigs compared with pellet-fed pigs in the present study (70% and 45% for mash and pellets in the present study, respectively). Therefore, we hypothesize that with higher occupancy rates, the hierarchy of the pigs within a pen may limit the FBHs of low-ranking pigs. 

## 5. Conclusions

To better understand and characterize the long-time evolution of the feeding behaviour habits of group-housed growing–finishing pigs, this work introduces a new approach (maintenance), which provides data at the individual level that are complementary to the classic repeatability concept. Broadly speaking, both approaches produced similar figures in the two experiments performed. Independently of the production conditions (environmental temperature and physical feed form), the repeatability and maintenance values of average daily feed intake progressively decreased as pigs grew. Only 50% of pigs maintained their average daily feed intake at the end of the growing–finishing period. By contrast, production conditions may modify the repeatability and maintenance of total feeder visits, total visit duration, feeder visit size and feeding rate and thus the whole feeding behaviour habits of group-housed growing–finishing pigs. Moreover, the maintenance approach is an interesting tool to use in other fields, such as ethology, in which the understanding of certain parameters at the individual level is of high interest, for example, when studying the influence of the hierarchy within a pen on the feeding behaviour habits of group-housed pigs. 

## Figures and Tables

**Table 1:** Schema of the pigs used in each trial.

Trials Analysed	Pigs Per Trial	Pens Per Trial	in Each Pen, Four Pigs of Each Body Weight Group
Hot-Temperate/Pelleted (HT-P)	60	5 pens of 12 pigs each	Light
			Medium
			Heavy
Temperate-Hot/Mash (TH-M)	60	5 pens of 12 pigs each	Light
			Medium
			Heavy

**Table 2:** Mean of the feeding behaviour habits by trial and period.

		Mean					
		p1 ^1^	p2 ^1^	p3 ^1^	p4 ^1^	p5 ^1^	p6 ^1^
HT-P ^2^	TVs ^3^, feeder visits/d	14.2	12.8	11.5	9.3	7.4	6.8
	TD ^4^, min/d	63.7	60.6	56.0	52.5	47.6	43.4
	VS ^5^, kg/feeder visit	0.115	0.143	0.182	0.248	0.316	0.361
	FR ^6^, g/min	23.7	27.9	33.6	39.5	46.1	52.3
TH-M ^2^	TVs ^3^, feeder visits/d	8.6	7.9	8.2	7.6	6.8	6.5
	TD ^4^, min/d	99.2	91.9	85.7	80.9	67.0	73.9
	VS ^5^, kg/feeder visit	0.187	0.225	0.246	0.324	0.295	0.380
	FR ^6^, g/min	15.2	18.4	23.9	28.3	28.7	32.6

^1^ Six periods of 14 days each (p1-p6) from day 31 to day 114 of fattening. ^2^ Hot-Temperate/Pelleted (HT-P) and Temperate -Hot/Mash trial (TH-M). ^3^ Total number of feeder visits per pig and day (TVs). ^4^ Total time spent eating per pig and day (TD). ^5^ Amount of feed intake per feeder visit (VS). ^6^ Feed intake per minute spent eating (FR).

**Table 3:** Trimmed mean and trimmed standard deviation of the feeding behaviour habits by trial and period. Standardized *z*-scores are obtained from these descriptive statistics.

		Trimmed Mean (Trimmed SD ^1^)					
		p1 ^2^	p2 ^2^	p3 ^2^	p4 ^2^	p5 ^2^	p6 ^2^
HT-P ^3^	TVs ^4^, feeder visits/d	2.6 (0.3)	2.4 (0.2)	2.4 (0.3)	2.2 (0.3)	1.9 (0.3)	1.9 (0.3)
	TD ^5^, min/d	4.1 (0.1)	4.1 (0.1)	4.0 (0.1)	4.0 (0.1)	3.8 (0.1)	3.8 (0.2)
	VS ^6^, kg/feeder visit	−2.198 (0.317)	−1.974 (0.276)	−1.753 (0.345)	−1.448 (0.365)	−1.207 (0.337)	−1.082 (0.358)
	FR ^7^, g/min	3.2 (0.2)	3.3 (0.2)	3.5 (0.2)	3.7 (0.2)	3.8 (0.2)	3.9 (0.2)
TH-M ^3^	TVs ^4^, feeder visits/d	2.6 (0.3)	2.4 (0.4)	2.4 (0.4)	2.2 (0.3)	1.9 (0.4)	1.9 (0.3)
	TD ^5^, min/d	4.1 (0.3)	4.1 (0.3)	4.0 (0.3)	4.0 (0.3)	3.8 (0.3)	3.8 (0.3)
	VS ^6^, kg/feeder visit	−2.198 (0.360)	−1.974 (0.419)	−1.753 (0.455)	−1.448 (0.391)	−1.207 (0.366)	−1.082 (0.325)
	FR ^7^, g/min	3.2 (0.3)	3.3 (0.3)	3.5 (0.3)	3.7 (0.3)	3.8 (0.3)	3.9 (0.3)

^1^ Trimmed Standard deviation. ^2^ Six periods of 14 days each (p1-p6) from day 31 to day 114 of fattening. ^3^ Hot-Temperate/Pelleted (HT-P) and Temperate-Hot/Mash trial (TH-M). ^4^ Total number of feeder visits per pig and day (TVs). ^5^ Total time spent eating per pig and day (TD). ^6^ Amount of feed intake per feeder visit (VS). ^7^ Feed intake per minute spent eating (FR).

## Appendix A

1. Example of usage
  To illustrate the complete procedure, consider the TH-M trial and two specific periods, *t = 1* and *t + 1 = 2,* as an example and take the parameter ADFI:
  1. Take *y_1_* = log (ADFI_1_) and *y_2_* = log (ADFI_2_), and let μ*_1,_*σ_1_, and μ*_2,_*σ_2_ be the respective trimmed means and standard deviations in the TH-M trial. Then, for each individual in this trial, calculate their z-scores in the two periods:
    $$z_{i1}=\frac{y_{i1}-\mu_{1}\;}{\sigma_{1}}\;\mathrm{and}\;z_{i2}=\frac{y_{i2}-\mu_{2}\;}{\sigma_{2}}$$
  2. Take a threshold δ, and compare the consecutive scaled positions; if $|z_{i1}-z_{i2}|\;\leq \;\delta$, we say that the analysed pig maintained the ADFI feeding behaviour at level δ, from period 1 to period 2;
  3. Finally, compute the maintenance value as a percentage of individuals whose measurements satisfy the inequality;
  4. The larger the maintenance value is, the better the reproducibility of FBHs (ADFI, in this example) is from one time interval to the subsequent one in this trial.

2. Technical note on the δ = ¾ threshold

Let Z_1_ and Z_2_ be the standardized scores representing a given feeding parameter in any pair of consecutive periods, and suppose that the scores fit a standard Gaussian distribution well. Moreover, assume that these two random variables are independent, that is, the maintenance of feeding habits is due to chance. In that case, $\frac{z_{1}-z_{2}}{\sqrt{2}}\sim N\left(0,1\right)\;$, and the probability of $|Z_{1}-Z_{2}|\;\leq \;\delta$ can be easily computed:

$$\pi_{\delta }=P\;\{|Z_{1}-Z_{2}|\leq \delta \}=2\cdot \Phi \;\left(\frac{\delta }{\sqrt{2}}\right)-1,$$

where Φ is the standard Gaussian cumulative distribution function. For $\delta =3/4,\;\pi_{\delta }\approx \;0.4$; therefore, a maintenance value of about 0.4 may be due to chance. On the contrary, a maintenance value over 0.51 is shown to be significant at the risk level of 0.05. Indeed, the upper tail bound for a confidence of 0.95 and a sample of size *n* = 58 is

$$\;\;\;\;\;\;\;\;\;\;\;\;\;\;\;\;\;\;\;\;\;\;\;\;\;\;\;\;\;\;\;\;\;\;\pi_{\delta }+z_{0.95}\;\sqrt{\pi_{\delta }\left(1-\pi_{\delta }\right)/n})\;\approx \;0.51,$$

where $z_{0.95}\;$ represents the standard Gaussian 95th percentile. Thus, a maintenance value of 51% or more is considered significant and due to feeding habit persistence. This is the case for TVs, TD, VS and FR, which show significant maintenance in all pairs of consecutive periods in both trials, whereas ADFI maintenance from p5 to p6 in both trials is not significant.

These technical arguments and the fact that repeatability and maintenance show congruent profiles are enough to consider δ=3/4  a reasonable choice.
